# Paramedian forehead flap combined with hinge flap for nasal tip reconstruction*

**DOI:** 10.1590/abd1806-4841.20164583

**Published:** 2016

**Authors:** Felipe Bochnia Cerci, Gerson Dellatorre

**Affiliations:** 1 Hospital Santa Casa de Curitiba – Curitiba (PR), Brazil

**Keywords:** Carcinoma, basal cell, Mohs surgery, Nose, Skin neoplasms, Surgical flaps

## Abstract

The paramedian forehead flap is a great option for restoration of complex nasal defects. For full-thickness defects, it may be used alone or in combination with other methods. We present a patient with a basal cell carcinoma on the distal nose treated by Mohs micrographic surgery, and a resulting full-thickness defect repaired with paramedian forehead flap combined with a hinge flap. For optimal results with the paramedian forehead flap, adequate surgical planning, patient orientation and meticulous surgical technique are imperative.

## INTRODUCTION

The paramedian forehead flap (PFF) is a single flap for restoration of complex nasal defects. It is the mainstay for reconstruction of large and deep wounds located on the distal nose (tip and ala), including full-thickness defects.^1,2^ The PFF can distinctively restore contour, texture, projection of the nasal tip and convexity of the ala, especially when combined with cartilage grafting.^1^ For full-thickness defects, PFF may be used alone or in combination with other methods to restore nasal lining.^3^

Prior to reconstruction, surgical margins should be completely evaluated by Mohs micrographic surgery (if available) since tumor recurrence beneath a PFF would be catastrophic.

## CASE REPORT

A 65-year-old man presented to the Department of Dermatology with a 1.8 x 2.0 cm erythematous nodule involving the nasal tip, left ala, soft triangles and collumela. Biopsy revealed an infiltrative basal cell carcinoma.

**Figura 1:**
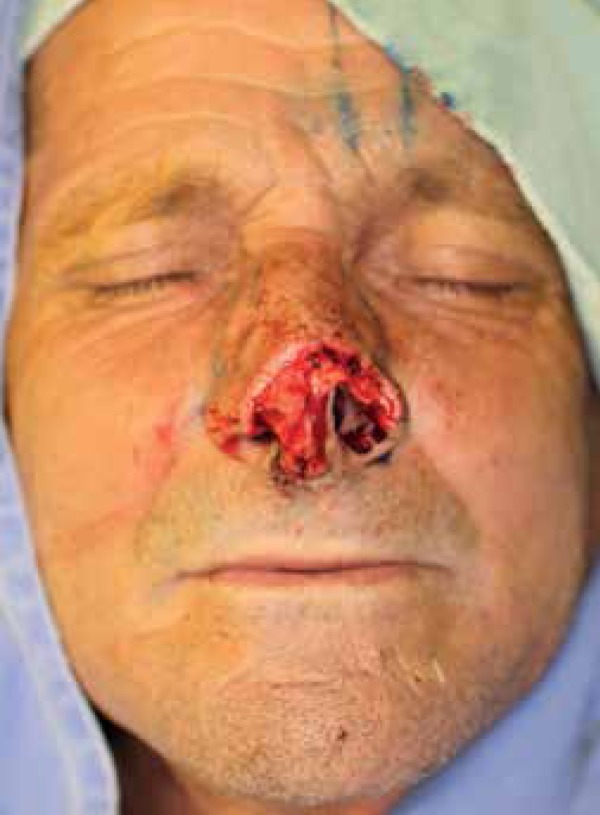
Surgical defect involving the nasal tip, nasal dorsum, left nasal ala, soft triangles and collumela.

The patient was submitted to Mohs micrographic surgery under local anesthesia (bupivacaine and lidocaine) combined with an oral benzodiazepine for additional comfort. After 3 stages, clear margins were achieved. The resulting defect measured 2.4 x 2.5 cm and affected the nasal tip, nasal dorsum, left ala, soft triangles and collumela (Figure 1). The lower lateral cartilage was partially removed and a portion of the defect had a full-thickness component. Due to the defect extension, it was opted to repair it with a PFF associated with a hinge flap.

**Figure 2:**
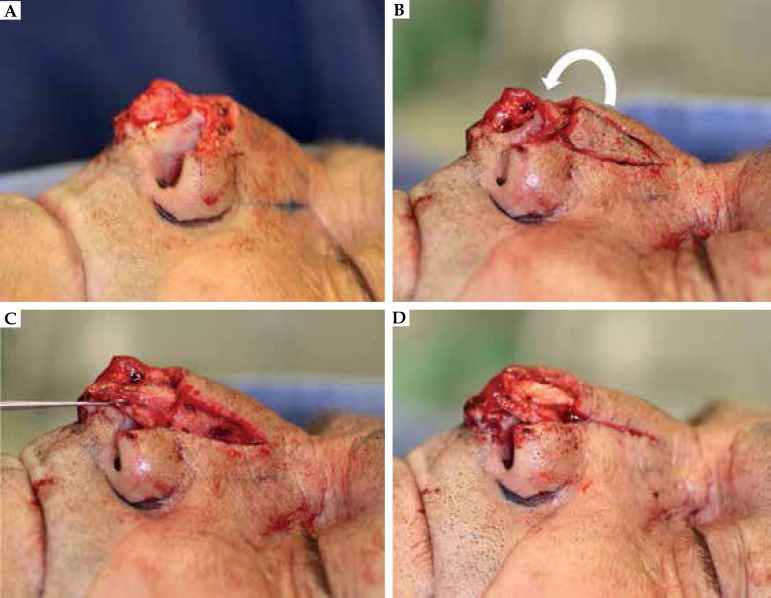
**A)** Full-thickness defect on the left nasal ala. **B)** Hinge flap incised on the left nasal sidewall and nasal dorsum. The arrow illustrates the 180 º movement to be performed by the flap. **C)** Flap positioned to recreate the nasal lining. **D)** Flap sutured in place. The nasal sidewall was closed primarily. Note cartilage graft sutured on the nasal tip.

Initially, in order to restore nasal lining, a hinge flap based on the nasalis muscle was performed from the nasal sidewall and nasal dorsum (Figure 2). Prior to suturing it in place, the flap was de-epithelialized. After that, the nasal sidewall was closed primarily. For better support and nasal tip projection, a cartilage graft was harvested from the left auricular concha through a posterior incision and sutured on the nasal tip.

**Figure 3:**
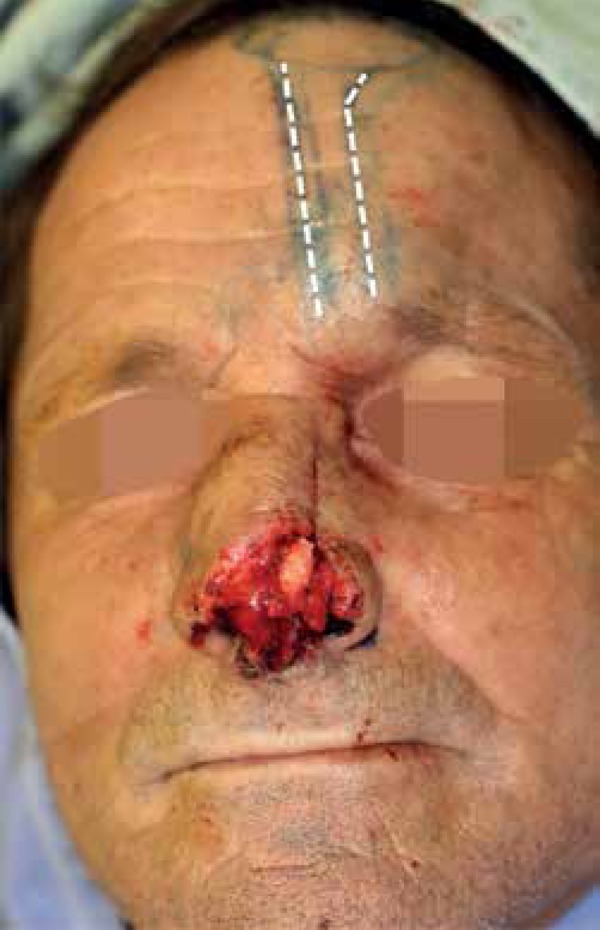
Paramedian forehead flap demarcated on the forehead with an ipsilateral pedicle (dashed) based on the left suprathroclear artery

After mucosal restoration and cartilage grafting, the PFF was designed based on the left suprathroclear artery. The pedicle (1.2 cm width) was demarcated 1.5 cm lateral to the midline. Then a template of the defect was made and demarcated on the forehead (Figure 3). The flap was then incised, elevated and sutured in two layers onto the primary defect. The forehead was closed primarily in three layers and its upper portion was left to heal by secondary intention.

**Figure 4:**
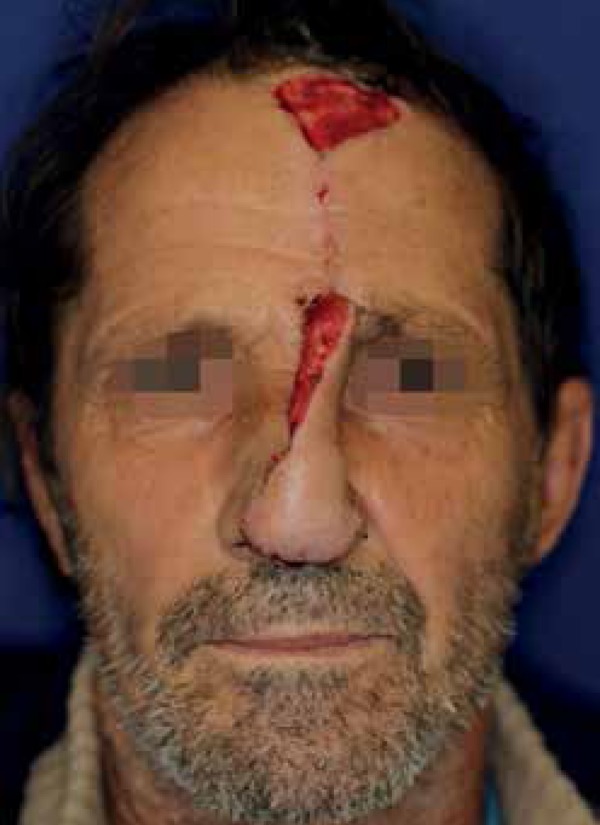
Three weeks after the first stage. Note the superior donor area on the forehead left to heal by second intention

**Figure 5:**
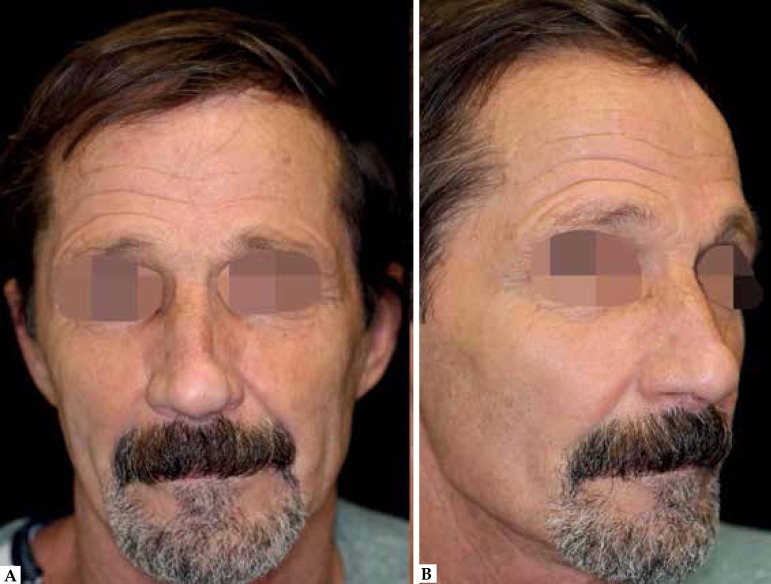
**A** and **B)** 4 months postoperatively with contour and texture restoration, and adequate nasal tip pro- A B jection

After three weeks, the patient was submitted to the second stage, which consisted of pedicle division and thinning of the proximal portion of the flap after careful elevation (Figure 4). Four months postoperative, the patient had a satisfactory result with nasal contour restoration and functional preservation, without complaints of breathing difficulties (Figure 5).

##  DISCUSSION

The nose is one of the most common locations for skin cancer and frequently represents a challenge for reconstruction after surgical defects. For larger defects on the distal nose, options that produce good functional and aesthetic outcomes are limited. When wounds are extensive, deep, and or involve missing cartilage or mucosal lining, no other repair can approach the consistency and predictability of the PFF.^1^ Full-thickness defects should be repaired in three layers: mucosal repair, cartilage grafting and soft tissue restoration.

PFF should be thought of as a robust surface covering that can provide soft tissue thickness but not structural support. Nasal lining and structural cartilage are the infrastructures that must be either intact, supplemented, and or restored prior to PFF.^4^ Options to restore small mucosal defects (<1cm) include a turnover hinge flap, turndown of a forehead flap extension, a full-thickness skin graft (FTSG), and bipedicle vestibular skin advancement flap. Larger lining restoration may require a turnover forehead flap, FTSG vascularized by an overlying PFF, or intranasal lining flaps (septal mucoperichondrial hinge flap, composite septal chondromucosal pivotal flap).^5,6^ Intranasal mucosal flaps are difficult to perform without sedation or general anesthesia. The other options mentioned above, however, may be successfully executed under local anesthesia. In the present case, a hinge flap from the nasal sidewall and nasal dorsum was performed due to the relative small size of the mucosal defect.

Cartilage grafts are either structural (native cartilage present but additional needed for support) or restorative (replacing what was removed). Structural functions of cartilage include: 1) preventing tissue contraction and distortion; 2) bracing heavy flap tissue; 3) maintaining airway patency and augmenting the internal nasal valve; and 4) achieving contour support (i.e. nasal tip graft for better projection).^1,2^ Donor sites for cartilage grafts may include the antihelix (scaphoid fossa) and the conchal bowl.^7^

PFF may be performed in two or three stages. For most patients, the two-stage approach is safely performed by debulking the distal portion of the flap in the first stage. As long as a thin layer of subdermal fat is preserved, then the supratrochlear artery is protected.^8^ The three-stage approach may be used for more complex defects or according to the surgeon´s preference.^9^

The forehead should be approximated as much as possible without tension. When significant tension is noted, the remaining wound should heal by second intention.^1,2^ The use of FTSG or STSG for the remaining donor site closure can result in a large “patchy scar”.

Potential complications of the PFF include bleeding, poor scarring, infection, dehiscence, distortion of free margins and flap necrosis. The safety of performing the PFF in an outpatient setting has been well documented, with low complication rates when performed with adequate technique.^10^

The PFF is a valuable flap in the repair of large and deep nasal defects following Mohs micrographic surgery. For full-thickness defects, a hinge flap should be considered to restore nasal lining. Good surgical planning, patient orientation and meticulous surgical technique are imperative for optimal results.
